# Elimination of Mother-To-Child Transmission of HIV Infection: The Drug Resource Enhancement against AIDS and Malnutrition Model

**DOI:** 10.3390/ijerph121013224

**Published:** 2015-10-21

**Authors:** Giuseppe Liotta, Maria Cristina Marazzi, Khethimipilo E. Mothibi, Ines Zimba, Evelyne E. Amangoua, Esther K. Bonje, Bernard N. B. Bossiky, Precious A. Robinson, Paola Scarcella, Kebby Musokotwane, Leonardo Palombi, Paola Germano, Pasquale Narciso, Andrea de Luca, Elard Alumando, Sangare H. Mamary, Nurja A. Magid, Giovanni Guidotti, Sandro Mancinelli, Stefano Orlando, Marco Peroni, Ersilia Buonomo, Karin Nielsen-Saines

**Affiliations:** 1 Department of Biomedicine and Prevention, University of Rome Tor Vergata, 18-00173 Rome, Italy; E-Mails: giuseppeliotta@hotmail.com (G.L.); paola.scarcella@gmail.com (P.S.); leonardo.palombi@gmail.com (L.P.); sandro.mancinelli@gmail.com (S.M.); ersiliabuonomo@gmail.com (E.B.); 2 Department of Human Sciences, LUMSA University, 18-00173 Rome, Italy; E-Mail: mcmarazzi@gmail.com; 3 Health Services Cluster, Cité du Djoué, P.O. Box 06 Brazzaville, South Africa; E-Mail: eula.mothibi@khethimpilo.org; 4 Community of S.Egidio—DREAM program, Avenida de Julho 7, Maputo, Mozambique; E-Mails: ineszimba@dream.org.mz (I.Z.); nurjamajid@yahoo.com (N.A.M.); 5 Ministère de la Santé et de la lutte contre le SIDA, B.P. 2091, Abidjan, Ivory Coast; E-Mail: evaehua@yahoo.fr; 6 Cameroon Baptist Convention Health Services, P.O. Box 1 Bamenda, Cameroon; E-Mail: kunibonje@yahoo.com; 7 Programme National Multisectoriel de Lutte contre le Sida, Blvd Triomphal and 24 Novembre Kinshasa, Congo (RDC); E-Mail: bernardbossiky@yahoo.fr; 8 National Department of Health, Private Bag X9070, South Africa; E-Mail: robinsonprecious@gmail.com; 9 Ministry of Health, Ndeke House, P.O. Box 30205, Lusaka, Zambia; E-Mail: paola.scarcella@gmail.com; 10 INMI L. Spallanzani, 00149 Rome, Italy; E-Mails: paolagermano1@gmail.com (P.G.); pasquale.narciso@inmi.it (P.N.); 11 Division of Infectious Diseases, Department of Medical Biotechnologies, University of Siena, Siena University Hospital, Siena 53100, Italy; E-Mail: andrea.deluca@unisi.it; 12 Community of S.Egidio—DREAM program, P.O. Box 30355, Blantyre, Malawi; E-Mails: eladarmando@yahoo.com (E.A.); drsangarehawa@gmail.com (S.H.M.); 13 Community of S.Egidio—DREAM program, Rome 00153, Italy; E-Mails: gianniguidotti1@gmail.com (G.G.); stefano.orlando@dreameurope.org (S.O.); peronimarco@gmail.com (M.P.); 14 Department of Pediatrics, David Geffen School of Medicine, University of California at Los Angeles, Los Angeles, CA 90095, USA

**Keywords:** elimination of HIV MTCT, DREAM program

## Abstract

The Drug Resource Enhancement against AIDS and Malnutrition Program (DREAM) gathered professionals in the field of Elimination of HIV-Mother-To-Child Transmission (EMTCT) in Maputo in 2013 to discuss obstacles and solutions for the elimination of HIV vertical transmission in sub-Saharan Africa. During this workshop, the benefits of administrating combined antiretroviral therapy (cART) to HIV positive women from pregnancy throughout breastfeeding were reviewed. cART is capable of reducing vertical transmission to less than 5% at 24 months of age, as well as maternal mortality and infant mortality in both HIV infected and exposed populations to levels similar to those of uninfected individuals. The challenge for programs targeting eMTCT in developing countries is retention in care and treatment adherence. Both are intrinsically related to the model of care. The drop-out from eMTCT programs before cART initiation ranges from 33%–88% while retention rates at 18–24 months are less than 50%. Comprehensive strategies including peer-to-peer education, social support and laboratory monitoring can reduce refusals to less than 5% and attain retention rates approaching 90%. Several components of the model of care for reduction of HIV-1 MTCT are feasible and implementable in scale-up strategies. A review of this model of care for HIV eMTCT is provided.

## 1. Introduction

Over the last two decades, the HIV epidemic in sub-Saharan Africa triggered a widespread reversal in health benefits which, prior to HIV, had been significantly improving in preceding years. The ensuing dramatic decline in life expectancy in countries with high HIV prevalence was demonstrative of the devastation caused by HIV/AIDS in the African continent. Mozambique was particularly stricken and ill-prepared for the rise of HIV/AIDS, as 26 years of war had just ended and the health care system was fragmented; funds for rebuilding the country were unavailable when HIV reached the population. HIV/AIDS was the tip of the iceberg in this scenario, as it enhanced underlying major health issues such as malnutrition, tuberculosis, malaria, gastrointestinal disease, and already-inflated alarming health indices, such as maternal and childhood mortality. Mozambique faced daunting challenges, including the restructuring and rebuilding of its health care system with very limited resources available to address the rising pandemic. Consequently, in the early 2000s the HIV epidemic found fertile ground in this environment. HIV-1 prevalence rapidly increased throughout the country facilitated by the Beira corridor (which linked Mozambique to Zimbabwe) in Central Mozambique, and the proximity of Mozambique to South Africa. 

The population of Mozambique is presently estimated at 27 million people, with women comprising 51%. Although Mozambique has achieved political stability and rapid economic growth in recent years, the HIV/AIDS pandemic represents a major obstacle to successful development. Mozambique describes its HIV epidemic situation as severe [1]. The UNAIDS Report on the Global AIDS Epidemic 2013 estimated that in 2012, an estimated 1.4 million individuals were living with HIV in the country including 6.6% of females and 2.8% of males age 15 to 24 years. The number of new infections in Mozambique in 2013 was estimated to be 130,000 [2]. Deaths due to AIDS in 2012 were reported at 77,000, with the number of HIV-infected pregnant women reported at 94,000 that year, and the number of new HIV infections in children estimated at 14,000 [2]. UNAIDS data demonstrated that, although in some African countries HIV incidence was declining (Ethiopia, Nigeria, Botswana, South Africa), in Mozambique the epidemic seemed to be levelling off at unacceptably high levels [3]. 

One of the key components to HIV prevention is EMTCT (Elimination of Mother to Child Transmission). It has been demonstrated to be not only highly effective but extremely beneficial to the promotion of maternal child health [4]. UNICEF reports that approximately 85 infants are infected daily with HIV in Mozambique [5]. As the overall risk of HIV MTCT in the first 12 months of age is approximately 35 to 40% in the absence of any intervention when *in utero*, *intrapartum* and breastfeeding components of transmission are combined [6], unidentified HIV-infected mothers carry a substantial risk of transmission to their infants, as well as their partners. In cases of acute maternal infection during pregnancy or in the postpartum period while breastfeeding, acute viremia prior to development of an adequate immune response increases MTCT risk substantially [7]. In resource-limited settings, half of the infants who contract HIV from their mothers die before their second birthday [8]. However, these deaths are preventable, as multiple studies in both developed and developing country settings have demonstrated. Early infant HIV-diagnosis accompanied by prompt antiretroviral treatment and prophylaxis of opportunistic infections can significantly reduce morbidity and mortality associated with HIV disease [9,10,11]. 

In Mozambique, HIV voluntary counselling and testing (VCT) efforts have been steadily increasing: in 2009 nearly one in two women (47%) underwent HIV VCT in antenatal clinics [1]. Although this represents a significant increase in comparison to a 3% 2003 HIV VCT rate in the country years before, it was still considerably lower than HIV VCT rates in pregnancy reported in neighboring countries such as Botswana (95%) or Namibia (64%) [12]. It is clear that accelerated EMTCT activities are warranted in Mozambique to enable the global attainment towards the elimination of new infections in children [13]. UNAIDS reported that referral and links of service between antenatal clinics and EMTCT are suboptimal in Mozambique, contributing to the drop out of women and children from EMTCT programs [13]. This includes poor social communication in the community, with a lack of psychological support for women recently identified as HIV-positive. This, in turn, contributes to the relatively low uptake of EMTCT services and a high drop-out rate for affected women and infants. Access to general health services, the main referral point for EMTCT, is also low, which enhances the bottleneck, limiting appropriate referrals. The links between pediatric treatment services and EMTCT services, which need to go hand-in-hand for a successful approach, are not optimized, and these need to be significantly strengthened in Mozambique. In summary, there is a need for an innovative model of care to address insufficient access to ART, lack of adequate facilities, deficiencies in training, and unavailability of health care workers which are the main limitations towards an adequate response to prevent HIV perinatal transmission.

In the present review, we aim to report our twelve year experience in the implementation of our DREAM HIV EMTCT model of care in Mozambique, with the purpose of describing how we developed and evolved a specific model of care for the elimination of mother-to-child HIV transmission at our centers. This model of care is highly applicable not only to Mozambique but to surrounding nations in sub-Saharan Africa and is presently being incorporated into practice by many neighboring countries. The objectives of this review are to report major challenges faced in the implementation of our EMTCT program over time and describe potential solutions to these multiple issues which were developed as our program evolved, resulting in what we believe is a successful implementation strategy. The purpose of the present manuscript is to describe the implementation of our program and highlight the pillars that sustain this type of approach so that our experience can assist with implementation of similar programs in other communities in sub-Saharan Africa.

## 2. Methods (Implementation of the DREAM eMTCT Program in Mozambique)

**Figure 1:**
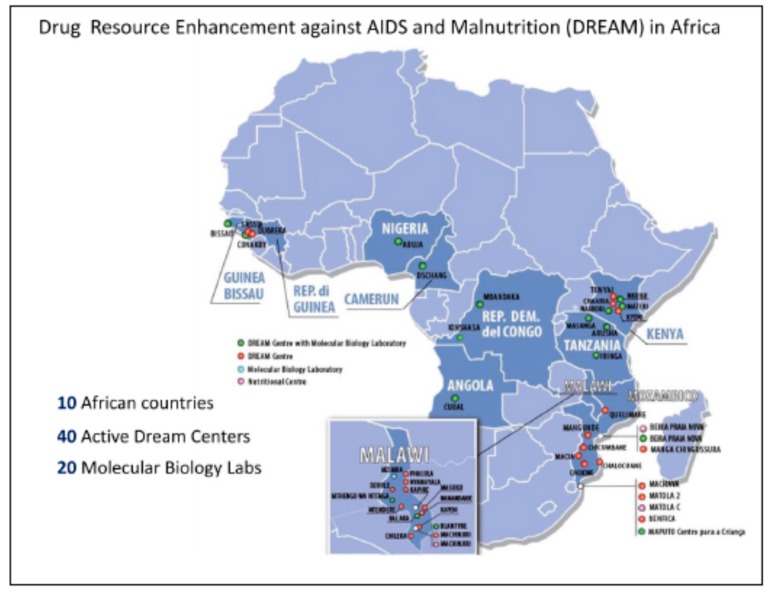
DREAM centers in Sub-Saharan Africa.

In 2002 the Drug Resource Enhancement Against AIDS and Malnutrition Program (DREAM) was created by a multidisciplinary team of medical professionals from the Community of Sant’Egidio, an Italian faith-based organization which had been in Mozambique for years promoting peace negotiations which culminated in the end of the civil war. DREAM, which began as a holistic public health program for provision of HIV care in Mozambique subsequently expanded within Mozambique and to nine other Sub-Saharan countries (Figure 1). The purpose was the development of a sustainable medical/ educational public health program for management, treatment and prevention of AIDS and malnutrition in sub-Saharan Africa. Subsequently the program became a constant partner of the Mozambican Ministry of Health’s HIV-driven initiatives and a collaborator of the United States President’s Emergency Plan for AIDS Relief (PEPFAR). Over the years, DREAM established a successful HIV program in Mozambique, with 73,000 cumulative patients followed including 55,700 on HAART (Highly Active Antiretroviral Therapy) under the auspices of PEPFAR. The DREAM HIV Prevention of Mother-to-Child Transmission (EMTCT) program in Mozambique advanced prevention efforts, with 16,200 HIV-infected pregnant women followed in DREAM centers over time. Some of the major obstacles identified by scaling-up initiatives included successful uptake of EMTCT interventions, retention of mother-infant pairs in EMTCT programs, and development of successful interventions geared towards reduction of HIV seroconversion in pregnancy or breastfeeding, as a large proportion of infant HIV-1 infections in Mozambique occur during this period [14]. 

The aim of DREAM has been to combine both prevention and treatment of HIV/AIDS in sub-Saharan Africa. Key elements include: [1] clinical care associated with access to standard and molecular laboratory diagnostics; [2] care free of charge to maximize access to services; [3] focus on the individual receiving care; [4] provision of nutritional support; and [5] informatization of data with electronic medical records in order to minimize errors and improve retention in care through an easier and faster identification of missed appointments and prompt access to medical information. 

In order to assess the successful implementation and uptake of our program in Mozambique we evaluated the following indicators following implementation: (1) number of pediatric and adult patients followed by year; (2) number of patients receiving antiretroviral treatment over time; (3) number of pregnancies and births followed in our program over time; (4) infant HIV-free survival; (5) maternal mortality; (6) population virus load and immune reconstitution; and (7) retention in care.

## 3. Results (Description and Outcomes following DREAM Program Implementation in Mozambique)

Our program presently provides HIV care to 300,000 individuals in Africa, over 150,000 are presently receiving Cart (Combination antiretroviral therapy). DREAM has presently over 12,000 HIV-infected children receiving cART, one of the largest pediatric cohorts receiving treatment in the continent. DREAM laboratories process over 60,000 patient specimens per year, for a cumulative total of 1,000,000 laboratory tests, including hematology, biochemistry, CD4 cell counts, and HIV-1 viral load determination. Dedicated computer systems and specific software constitute an important support to these activities. As a result of the DREAM program implementation in sub-Saharan Africa, particularly in Mozambique, approximately 10,000 African health care workers including medical doctors, nurses, biologists, laboratory technicians, center coordinators/administrators, home care assistants, and computer experts have participated in 24 Pan-African training courses organized from 2002–2014.

**Figure 2:**
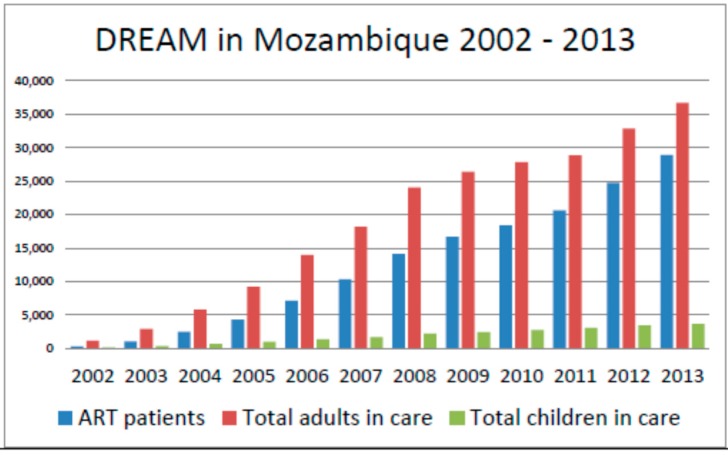
Patients receiving care at DREAM centers in Mozambique over the years.

**Figure 3:**
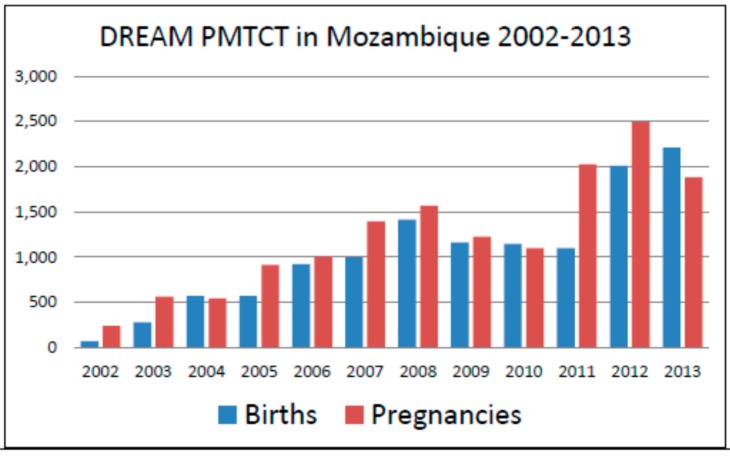
HIV+ pregnancies and HIV-exposed infants followed in DREAM centers in Mozambique over time.

The DREAM program in Mozambique manages 10 heath care facilities in the country, both in urban and rural settings, with care provided to over 35,000 HIV-infected patients on cART (Figure 2 and Figure 3). The program is particularly focused on HIV EMTCT [15,16,17,18,19]. Growth of the program steadily increased from inception in 2002 to 35,000 patients followed in 2013, an indicator of successful program uptake by individuals in the country. The number of pregnancies in HIV-infected mothers followed at our centers has steadily risen over the years as seen in Figure 3, another marker of successful uptake. Our infant HIV-1 free survival at 12 months of age of HIV-exposed infants born to women receiving HIV care during pregnancy at DREAM centers is 92.5%, which is in stark contrast to infant HIV-free survival nationwide [15]. Given that in the absence of any HIV EMTCT intervention, a 30%–40% HIV transmission rate would be expected [6], MTCT rates of 2% or less in a breastfeeding population [15,16,17,18,19] are highly encouraging, particularly since most HIV EMTCT programs in Mozambique have demonstrated HIV mother-to-child transmission rates of 5%–10% or higher in recent years [20].

In recent years the DREAM program and Mozambican government initiated combined scale-up activities in preparation for universal antiretroviral treatment of all pregnant women. This included the expansion of the use of triple antiretroviral therapy for prevention of HIV vertical transmission; a strategy for countrywide implementation of nutritional supplementation to HIV-infected pregnant women and their children; expansion of laboratory diagnosis and monitoring; formation and training of clinicians in the area of HIV/AIDS; development of institutional support systems for health information; and development of strategies for universal access to antiretroviral therapy. The agreement proposed the inclusion of strong community involvement, a major feature of the DREAM program [21,22]. 

A major priority in the model proposed by our program is treatment of pregnant women and the mother-infant pair. While therapy for individuals for HIV/AIDS aims to counter the progression of infection, and the program has also targeted this objective [22,23,24,25,26], treatment of mothers not only prevents a new infection but safeguards the infant’s health. DREAM program studies, and those of others, have demonstrated that maternal health is directly associated with infant health [15,27,28], a reason why maternal health is one of UNICEF’s Millennium Development Goals [28]. In the DREAM EMTCT program, HIV-infected women are offered cART as of the 14th week of gestation, if needed for their own health (CD4 cell count less than 350 cells/mm^3^ or WHO clinical stage 3/4) or as of the 25th week of pregnancy if prescribed for EMTCT purposes. Following delivery, all women continue HAART while breastfeeding (even if clinical, immunologic or virologic eligibility criteria for HAART are not yet met). This therapeutic approach has led to highly successful infant and maternal outcomes [15,16,17,18,19,27,29,30]. 

**Figure 4:**
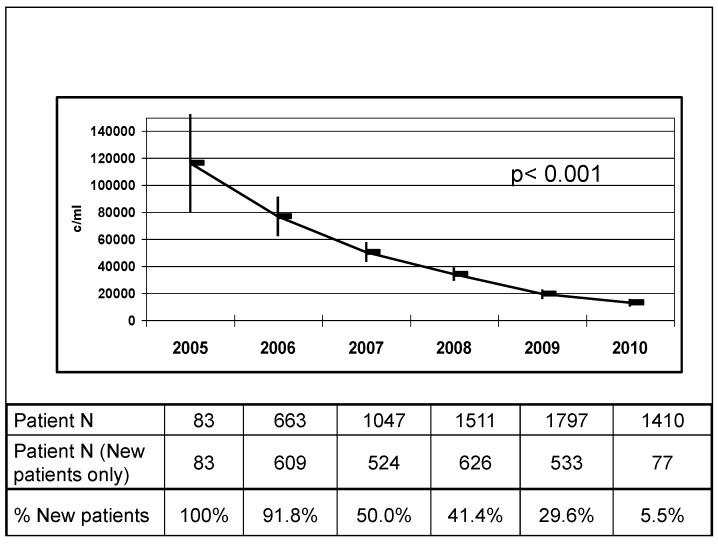
Mean virus load (and 95% CL) of DREAM program pregnant patients at one of our centers over time. CL: Confidence Limits.

Our program saw a very significant increase over time in the total number of patients followed in Mozambique, both adult and pediatric, and the number of subjects prescribed cART as seen in Figure 2. The number of pregnancies monitored through our services also significantly increased over the years as seen in Figure 3. We documented in numerous publications a steady decrease over the years in HIV mother-to-child transmission rates among our patients, from 5% in earlier years to less than 2% at 12 months of age [15,17,18,19]. Our infant HIV-free survival at 18 months has been 92.5% or higher in multiple studies [15,16,17,18,19], a result of implementation of a WHO B type approach in our treatment protocols years before this practice became standard of care endorsed by the WHO. The implementation of cART to all pregnant women regardless of CD4 cell count highly impacted short-term and long-term maternal mortality rates in our postpartum population of women. Among 10,150 pregnancies of HIV-positive women followed at our centers, long term maternal mortality (up to four years post-delivery) was 2.3%, [31] which is significantly less than maternal mortality rates observed in sub-Saharan Africa, where maternal mortality can reach 500 deaths per 100,000 births or 1 in 39 [32]. Maternal mortality was particularly reduced in women who received long courses of antenatal cART in our cohorts, demonstrating that the benefits of cART for EMTCT extend way beyond transmission efforts but also reduce maternal deaths [31]. The implementation of widespread cART to our patients has also been associated with significant declines in population virus load parameters and immune reconstitution, with significant gains in CD4 cell count numbers and restoration of immune function [23,25]. As Figure 4 illustrates, mean virus load values of pregnant patients followed in our clinics has steadily decreased over the years, likely a reflection of widespread use of cART in our patient population.

Finally, retention in care of pregnant and lactating mothers has been enhanced by the provision of cART during breastfeeding at our centers [21]. One of our major efforts has been towards prompt initiation of ART as early as possible during pregnancy, with a median time of cART initiation during pregnancy of 14 days from initial consultation for HIV prenatal care in 1830 women followed in one of our studies [33]. The implementation of specific software to our electronic medical records to track attrition in our patient population enables our clinics to proactively monitor patient adherence and retention [33]. These performance indicators have enabled the continued assessment of our EMTCT program implementation over the years. 

## 4. Discussion: Challenges and Potential Solutions

In recent years, the WHO introduced a triple-therapy option for HIV-positive pregnant women targeting both prevention of transmission to children and appropriate treatment of mothers (Maternal triple ARV prophylaxis to prevent MTCT, Option B), or antiretroviral treatment for life, Option B+. [34]. Infant prophylaxis in our program consists of single dose nevirapine provided as close as possible to birth, within 72 hours of life. Infants are evaluated shortly after birth with basic anthropometric parameters recorded. If infants have a low birth weight or demonstrate any other clinical problem, frequent clinical examinations are scheduled. 

A key element of the program is early virologic diagnosis of infants, so that prompt cART may be initiated if necessary to decrease morbidity and mortality risk. DREAM centers are supported by their own molecular biology laboratories which perform HIV DNA PCR testing of infant specimens by trained laboratory personnel from Mozambique. HIV b-DNA (Siemens) or PCR (Abbott) testing of infants at one, six, and 12 months of age and HIV-1 serologies at 18 months are routinely performed. 

Nutritional supplementation: The program offers nutritional supplementation to all pregnant and lactating HIV-infected mothers followed at our centers as malnutrition and anemia are very significant problems in Mozambique, even in the absence of HIV infection. As such, nutritional supplementation has been a key component of the program, not only for retention in care but also for improved health outcomes [35,36]. Throughout pregnancy and until the child is 18 months old, patients are guaranteed monthly food support (rice or maize, beans, peanuts, oil, sugar) and multivitamin supplementation. This measure counteracts malnutrition and anemia and contributes to the reduction of premature deliveries and low birth weight [15,16,20,26,35]. In the postpartum period, nutritional supplementation also assists with infant development following weaning and contributes to maintaining adequate maternal caloric intake while breastfeeding [15,35,36,37]. Maternal antiretrovirals are continued until HIV-exposed infants are completely weaned. For this purpose breastfeeding mothers are encouraged to participate in nutritional classes so that they can be prepared and educated about the weaning process. Nutritional supplementation to patients with reduced BMI or an AIDS diagnosis is also provided. 

Diagnostics: Diagnostic capabilities are necessary for a successful HIV prevention and treatment initiative, including the ability to monitor immunologic function through CD4 cell count assessments (flow cytometery) and virus load, as well as basic hematologic and biochemistry panels. Molecular laboratory techniques have been used routinely for early diagnosis of infants as well as measurement of virus load for therapeutic monitoring in the DREAM program. This was implemented since the start of the program, which entailed a completely new approach to diagnostics and monitoring in this setting [15,16,17,18,19,24,25]. 

**Figure 5:**
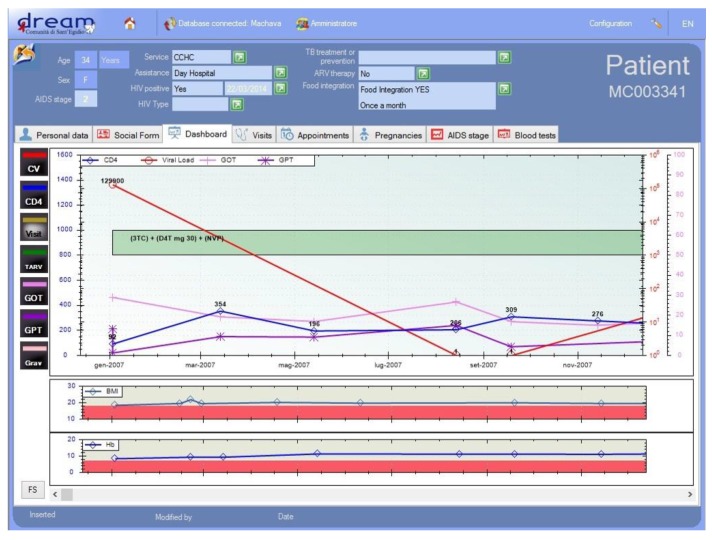
Electronic medical records.

Electronic medical records: Use of electronic medical records is routine throughout the program centers with informatization of all patient data, including medical history and physical exams, laboratory data, appointments, medical encounters, prescription refills, and nutritional supplementation. This enables caretakers to plot virus load data, CD4 cell counts and body mass index data in real time while the patient is being evaluated as well as monitoring of adherence to prescriptions and medical visits [38] (Figure 5). 

Community activists and their role in EMTCT uptake and reduction of HIV-associated stigma: Community-based strategies which effectively increase EMTCT uptake and reduce HIV-stigma are compatible and feasible with program scale-up not only in Mozambique but in sub-Saharan Africa as a whole. The partnering of new patients with established patients who guide the new ones through the process and follow-up on routine procedures such as registration, appointments, and initiation of antiretroviral therapy, including checking that prescriptions have been filled at pharmacies has been proven to be very successful for retention in care. The use of community participants who support uptake of EMTCT care is a crucial step in the DREAM program which ensures that patients referred to our facilities are not lost through the referral process or “fall through the cracks” in a system they are not familiar with [20,21,35]. The use of peer activists has been a significant step towards reduction of stigma. 

Community and health professional education: Referrals to EMTCT care are dependent on caretaker and community awareness of the HIV problem. The DREAM program formed a network of medical professionals who work in conjunction with a large patient network for education of the community and awareness of EMTCT issues. Community activists organize monthly events including campaigns and festivals for patient networks and also for the overall community to increase HIV EMTCT awareness. One of the most significant challenges faced by countries affected by the HIV pandemic in sub-Saharan Africa is the lack of skilled human resources and medical professionals trained to address multiple HIV and infectious diseases issues. In order to generate a solid base of trained health care workers and community health workers who can work with HIV, the program developed Pan-African HIV courses which are attended by approximately 250 medical professionals and community health care workers from multiple African countries, including health care workers who are not necessarily working for the organization. Tele-medicine capabilities are often used to enhance participation. The goal is to improve the quality and knowledge of health care workers in this setting, as successful HIV care services depends on well trained providers. 

Retention in Care: Specific interventions which increase retention in care of HIV-infected pregnant women and their infants in the first 12 months postpartum are much needed in sub-Saharan Africa. A key component for retention in care of expectant mothers is delivery of hope to patients. This is made possible through the provision of HAART in a timely manner to expectant mothers in association with counselling for adherence provided by community based members. Provision of medicines that effectively treat HIV reassure patients that their condition is treatable and that everything possible is being done to protect their infants from acquiring HIV. Patient education and awareness of HIV signs and symptoms is also critical for engagement and retention. For this purpose, besides the frequent support meetings provided to patients through a community network, there is also provision of a variety of educational materials including books and pamphlets published in local dialects for patient education. If patients consent, community members perform home visits for patient education and evaluation, assisting them with prescriptions and intake of medications. While patients receive antiretrovirals during pregnancy and in the postpartum period, this network of support for adherence is established, and retention in care is significantly facilitated. The experience of the program has been that once antiretrovirals are stopped because women are no longer breastfeeding, retention in care often becomes a problem.

Prevention and early identification of incident HIV infections in pregnancy: One specific problem in Mozambique which heavily affects the country’s PMCT efforts is the prominent issue of HIV seroconversion during pregnancy or breastfeeding [14,39]. In general HIV-1 VCT in antenatal clinics in Mozambique is performed once during prenatal care, and not routinely repeated. Particularly in the Maputo region, where partners and spouses may be employed as miners in neighboring South Africa, incident HIV infections have been reported at very high rates. In a country with an overall 15% HIV prevalence in pregnancy, this translates into approximately 14,000 incident infections in pregnant or lactating women, who would not be otherwise identified, thus resulting in approximately 4500 infant infections per year. As seroconversion during pregnancy is a major risk factor for HIV MTCT [39], for prevention efforts to be broadly successful the issue of incident HIV infections in this population needs to be addressed. 

Maternal mortality and protective effects on infant survival in DREAM: In a recently published paper, 10,150 pregnancies followed in the DREAM program were described [31], with a 1% maternal death rate up to six weeks postpartum. The study showed an increased mortality in patients with shorter antenatal HAART: 2.2% if less than 30 days and 0.9% if 31 days or greater (*p* < 0.001). Four years later, survival was 92% for women with shorter antenatal HAART and 98% for women on established therapy prior to pregnancy, *p* = 0.001. ART in pregnancy has a further added value, being not only powerful in preventing vertical transmission but also highly protective for survival of mothers. A previous study [15] based on a cohort of 3071 pregnancies resulting in 3148 live births showed a protective effect also on infant outcomes. Infant HIV-1-free survival at 12 months was 92.5%. Mother-to-child transmission and/or infant deaths correlated with length of maternal antenatal ART by multivariate analysis at one, six, and 12 months. The study demonstrated that extended antenatal ART is protective against adverse infant outcomes up to 12 months of age even in children born to mothers with higher CD4 cell counts.

### Cost-Effectiveness of the DREAM Program

The DREAM EMTCT approach is complex, but it has also proven to be cost-effective [40]. In a study published in 2010, results of the DREAM model in Malawi were compared to a no intervention scenario from an economic point of view. Although more expensive, the cost of the DREAM EMTCT approach was largely compensated by the benefit provided through the reduction in the numbers of HIV-infected children in the community and the health related costs averted, not to mention the loss of economic productivity to future generations. 

## 5. Conclusions

One of the lessons learned from the DREAM experience in sub-Saharan Africa is that for retention in care and adherence to regimens to take place, the general model of care through which HIV treatment and prevention of vertical transmission are delivered should be updated for the region. For programs to be successful, they should be (1) free-of charge; (2) have a secure supply chain for rapid HIV test kits and antiretrovirals availability; (3) implement opt-out strategies for HIV counseling and testing; (4) deliver a comprehensive holistic approach to the family unit which will drive the elimination of MTCT efforts; (5) integrate both HIV EMTCT and ART services within antenatal services; (6) incorporate strategies which enable mother to easily access EMTCT efforts, such as task shifting, and mobile clinics in rural areas; (7) have community support for continued adherence to EMTCT programs through community health workers trained for this purpose; (8) have the availability of early infant diagnosis (EID) and reasonable turn-around times for results to be delivered for HIV-exposed infants as the availability of EID encourages mothers with the knowledge that their infants are negative, or otherwise provides reassurance that early treatment to HIV-infected babies is available; (9) provide nutritional assessment and assistance to mothers and infants; (10) undergo continuous monitoring and evaluation as timely supervision with quality assurance is crucial for the assessment of the impact and quality of programs; and (11) implement information technology to support care and guarantee timely tracing of lost-to-follow up, which will enhance retention. 

The next steps towards elimination of HIV mother-to-child transmission in sub-Saharan Africa is the development of a comprehensive model for enhanced retention in HIV EMTCT programs in the continent, in order for countries to reach the target of zero new infections in the future. The DREAM group of investigators, convened in Maputo for a review of the program and its results, strongly endorsed a unified effort across sub-Saharan Africa to enable the systematic inclusion of these elements into an HIV elimination of mother-to-child transmission model. 
